# Globus Pallidus Externus Neurons Expressing *parvalbumin* Interconnect the Subthalamic Nucleus and Striatal Interneurons

**DOI:** 10.1371/journal.pone.0149798

**Published:** 2016-02-23

**Authors:** Arpiar Saunders, Kee Wui Huang, Bernardo Luis Sabatini

**Affiliations:** Department of Neurobiology, Howard Hughes Medical Institute, Harvard Medical School, Boston, Massachusetts, United States of America; Florey Institute of Neuroscience and Mental Health, The University of Melbourne, AUSTRALIA

## Abstract

The globus pallidus externus (GP) is a nucleus of the basal ganglia (BG), containing GABAergic projection neurons that arborize widely throughout the BG, thalamus and cortex. Ongoing work seeks to map axonal projection patterns from GP cell types, as defined by their electrophysiological and molecular properties. Here we use transgenic mice and recombinant viruses to characterize *parvalbumin* expressing (PV^+^) GP neurons within the BG circuit. We confirm that PV^+^ neurons 1) make up ~40% of the GP neurons 2) exhibit fast-firing spontaneous activity and 3) provide the major axonal arborization to the STN and substantia nigra reticulata/compacta (SNr/c). PV^+^ neurons also innervate the striatum. Retrograde labeling identifies ~17% of pallidostriatal neurons as PV^+^, at least a subset of which also innervate the STN and SNr. Optogenetic experiments in acute brain slices demonstrate that the PV^+^ pallidostriatal axons make potent inhibitory synapses on low threshold spiking (LTS) and fast-spiking interneurons (FS) in the striatum, but rarely on spiny projection neurons (SPNs). Thus PV^+^ GP neurons are synaptically positioned to directly coordinate activity between BG input nuclei, the striatum and STN, and thalamic-output from the SNr.

## Introduction

The basal ganglia (BG) are interconnected forebrain nuclei necessary for selecting and shaping motor and cognitive behaviors. BG circuits contain an assortment of cell types that mediate synaptic interactions within and between BG nuclei. The diversity and function of BG cell types is best understood in the striatum, which contains spiny projection neurons (SPNs) and a handful of distinct interneuron types[1]. Based on axonal projections[2], electrophysiological properties[3] and dopamine receptor expression[4], SPNs fall into two major categories. This subdivision is the basis for the prominent model explaining how the BG control cortical feedback and behavior[5,6]: direct pathway SPNs (dSPNs) promote actions by disinhibiting the thalamus and cortex, whereas indirect pathway SPNs SPNs (iSPNs) dampen or sculpt actions by indirectly disinhibiting the SNr and thus potentiating BG inhibitory outputs. Although simplistic in both connectivity[7,8] and coding[9,10], the pathway model does largely explain how SPN activity affects cortical firing rates[11] and motor behavior[12,13]. In patients with Parkinson’s or Huntington’s Disease, the degeneration of specific BG cell types results in distinct symptomatologies[14]. These results demonstrate that BG cell types play unique and vital roles in behavior and suggest that a comprehensive model of BG circuitry necessitates a complete description of intrinsic cell types.

The globus pallidus externus (GP), a central nucleus of the BG, was originally considered a simple relay within the BG [6]. However, the GP is one of most transcriptionally distinctive regions of the human brain[15] and a growing body of work has accelerated our understanding of how this molecular diversity maps onto distinct cell types. GP neurons are known to be GABAergic[16–18], spontaneously active *in vitro*[19] and *in vivo*[20] and project throughout the BG, including the subthalamic nucleus (STN), globus pallidus internus (GPi), substantia nigra (reticulata and compacta, SNr/SNc), and the striatum[21,22]. GP neurons also project to the reticular[23,24] and parafasicular[25] nuclei of the thalamus. Building on anatomical work [26,27], we and others recently characterized a GP projection to cortex [28,29]. This pallidocortical system consists of wide-spread axons originating from a small population of GABAergic neurons intrinsic to the GP proper and a larger set of Cholinergic/GABAergic projection neurons within and surrounding the GP border [27,28,30]. Like canonical GP cells, pallidocortical cell types are innervated by iSPNs and dSPNs of the dorsal striatum and glutamatergic inputs from STN. Thus GP neurons are positioned to respond to the major BG pathways and coordinate activity throughout the brain using synaptic inhibition[31,32].

Classic studies described a handful of GP neuron subtypes based on intrinsic membrane properties in brain slices[33–35] and firing patterns *in vivo* [20]. Recent work has made striking progress at mapping observed electrophysiological diversity to anatomical and molecular expression characteristics, especially those tracking developmental origin [25,36–38]. This molecular framework has focused on the canonical pallidostriatal and pallidosubthalamic projections. For example, approximately half of all GP neurons express the calcium binding protein parvalbumin (PV) in mice (29, 51, and 55%)[25,36,38] and rats (59–63%). [37,39]. Fate-mapping experiments in mice demonstrate that these PV^+^ cells originate from the ventral portion of the medial ganglionic eminence (MGE) and express the transcription factor Nkx2-1. PV/Nkx2-1^+^ neurons define a class called “Prototypic,” which innervate the STN and are the most abundant neuron population in in rodents [36,37]. Another distinct GP cell type, called “Arkypallidal,” originate from the caudal/lateral ganglionic eminence (CGE/LGE) and largely, though maybe not exclusively, innervate the striatum[36–38,40,41]. These cells make up about ~25% of the rodent GP and express the transcription factors FoxP2, Npas1 and the neuropeptide preproenkaphalin (PPE)[36,37]. Prototypic and Arkypallidal neurons are distinguished by their intrinsic and active membrane properties [37,38] and *in vivo* responses to movement [36], suggesting distinct circuit functions. Manipulations of dopamine or dopamine receptors exacerbate differences in Prototypic/Arkypallidal circuit activity [40], cell-autonomous firing [38], and immediate early gene expression [42], suggesting the striatal dopamine signaling differentially engages each cell type.

Not all molecular markers are strictly selective for Prototypic or Arkypallidal neurons, complicating the picture of neuronal diversity within the GP. For example, immunostaining for the LGE transcription factor Npas1 demarcates cells that largely, but perhaps not exclusively[38], innervate the striatum. Npas1 also appears to be expressed in a small subset of PV^+^ cells, one of the major markers of STN-targeting Prototypic neurons[37]. Note however, that this overlap was not observed in specific yet not fully penetrant Npas1 BAC transgenic line[38]. The MGE transcription factor Lhx6 also defies the Prototypic/Arkypallidal division. While one study found a lack of overlap between Lhx6 and PV expression using a BAC transgenic mouse[25], others have described Lhx6 in both PV^+^ and Npas1^+^ populations as well as an Lhx6^+^ group devoid of both PV/Npas1 expression[38]. Some of these molecular intersections may represent distinct cell types with unique cellular signaling and circuit functions. Others may represent discrepancies based on differences in methodology or uncontrolled aspects of biological states. Despite careful work and important advances, the extent to which molecular markers identify trends vs. rules of GP cell type connectivity remains unclear.

Current models do converge on the idea that PV^+^ Prototypic neurons directly control BG outputs by innervating the STN and SNr/c, whereas PV^-^ Arkypallidal neurons influence BG inputs through GABAergic innervation of the striatum. However, at least a subset of GP neurons innervate both the striatum and SNr, highlighting the oversimplified relationship between molecular markers and axon projection patterns[43].

Here we use recombinant viruses and transgenic mice to study the non-canonical PV^+^ projection from GP to striatum (GP-Str). We confirm that PV^+^ neurons make up around 40% of the mouse GP and exhibit faster firing rates than PV^-^ neighbors [25,37,38]. We show that PV^+^ neurons make up the minority of GP-Str projecting neurons (~15%), but selectively innervate fast-spiking (*PV*^+^) and low-threshold spiking (*Neuropeptide Y*^*+*^) striatal interneurons and the neurons of the STN. Since inhibiting interneurons could have a large effect on striatal output, PV^+^ GP neurons could play a unique role in BG circuits by directly coordinating the striatum and STN in a regime of fast-firing rates (20–80 Hz). PV^+^ GP-Str neurons may thus contribute to the inverse relationship in firing rates exhibited by striatal interneurons and the GP during behavior [44]. Our results provide important information about the circuit context of a non-canonical GP cell type.

## Materials and Methods

### Mice

Cre recombinase was targeted to *parvalbumin* (Gene ID: 19293) expressing cells of the GP and striatum using transgenic knock-in mice that link Cre expression to the parvalbumin locus with an internal ribosome entry site (*PV* ^i-Cre^). *PV* ^i-Cre^ were generated by the Arber lab [45] and obtained from the Jackson Labs (stock # 008069). Both *PV* ^i-Cre/+^ and *PV* ^i-Cre/ i-Cre^ mice were used for experiments. To visualize the full processes of Cre expressing cells, Cre mice were bred to Cre-dependent tdTomato reporter allele (Ai14; Jackson Labs, stock # 007914; referred to as Rosa26^lsl-tdTomato^). Striatal cell types were identified by a combination of neuronal morphology and molecular markers, including bacterial artificial chromosome (BAC) transgenes expressing EGFP under the control of the dopamine 2 receptor (*Drd2-*EGFP; GENSAT founder line S118) and Neuropeptide Y (*NPY-*EGFP; Jackson Labs, stock # 006417) regulatory sequence. Despite some differences in strains of origin, all transgenic mice were maintained on a predominantly C57BL/6 background. All wild-type mice were C57BL/6 and obtained from Charles River. All experimental manipulations were performed in accordance with protocols approved by the Harvard Standing Committee on Animal Care following guidelines described in the US National Institutes of Health *Guide for the Care and Use of Laboratory Animals*.

### Virus Preparation

Cre conditional expression was achieved using recombinant adeno-associated virus (rAAV) carrying transgenic cassettes whose transcription was activated or inactivated by Cre. Cre-activated (“Cre-On”) expression of channelrhodopsin-2 (ChR2-mCherry, H134R variant) or EGFP was achieved by using a double-floxed inverted open reading frame (DIO). To achieve simultaneous Cre-On EGFP and Cre-inactivated (“Cre-Off”) tdTomato labeling, rAAVs DIO-EGFP and FAS-tdTomato were mixed 1:1 [46]. DIO and FAS rAAVs all use the EF1α promoter and were packaged in serotype 8 by a commercial vector core facility (University of North Carolina), except for the DIO-TC^B^, which uses the CAG promoter and was packaged by the Boston Children’s Hospital vector core in serotype 9. CAG-Flex-TC^B^ was a gift from Liqun Luo (Addgene plasmid # 48332). All rAAVs were stored in undiluted aliquots at a concentration >10^12^ genomic copies per ml at −80°C until intracranial injection. An EnvA-pseudotyped, glycoprotein-deleted rabies virus carrying the EGFP transgene (EnvA-SADdG-EGFP) was generated in-house, using starting materials that were a gift from Byung Kook Lim (UCSD). The recombinant rabies viruses (rRV) were generated using BHK-B19G and BHK-EnvA cells using protocols similar to those previously described[47], and injected at a concentration of approximately 1.0 x 10^9^ infectious units/ml.

### Stereotaxic Intracranial Injections

Male and female mice (postnatal day 14–25) were anesthetized with isoflurane and placed in a small animal stereotaxic frame (David Kopf Instruments). Under aseptic conditions, the skull was exposed and a small hole was drilled. For rAAV injections, 200–250 nl total volume was delivered bilaterally into the GP through a pulled glass pipette at a rate of 100–200 nl·min^−1^ using a Microinject system (World Precision Instruments). GP injection coordinates were 0.5 mm posterior from Bregma, 1.9 mm lateral and 3.1 mm below the pia. The same technique was used to inject 100 nl of undiluted red retro beads (Red-1X, Lumafluor) or the rRV EnvA-SADdG-EGFP into dorsal striatum using the coordinates 0.9 mm anterior from Bregma, 2 mm lateral and 2.7 mm below the pia. After surgical procedures, mice received flunixin for analgesia and were returned to their home cage for >14 days allow for maximal gene expression or 4 days to allow for bead retrograde transport. Stereotaxic coordinates were adjusted slightly by age. For tracing experiments using the rabies virus, AAV-CAG-DIO-TC^B^ or AAV-EF1α–ΔIO-mCherry was injected bilaterally into the GP and allowed to express for >14 days before EnvA-SADdG-EGFP was injected bilaterally into the dorsal striatum. Mice were sacrificed 5 days after the injection of the rabies virus for histological analysis.

### Tissue Processing, Immunohistochemistry & Cell Counting

Mice were deeply anesthetized with isoflurane and transcardially perfused with 4% paraformaldehyde (PFA) in 0.1 M sodium phosphate buffer (1x PBS). Brains were post-fixed for 1–3 days, washed in 1x PBS and sliced into sagittal or horizontal sections (40 μm) using a Vibratome (Leica). For Parvalbumin and NeuN immunohistochemistry, slices were incubated in a 1x PBS blocking solution containing 5% normal horse or goat serum and 0.3% Triton X-100 for 1 hour at room temperature. Slices were then incubated overnight at 4°C in the same solution containing anti-Parvalbumin (1:1000, Millipore MAB1572) or anti-NeuN (1:100, Millipore MABN74) primary antibodies. The next morning, sections were washed 3 times for 5 minutes in 1x PBS for and then incubated for 1–2 hours at room temperature in the blocking solution containing goat anti-mouse Alexa 594 or goat anti-rabbit Alexa 488 (1:500, Molecular Probes). Slices were then mounted on slides (Super Frost) and allowed to dry before coverslipping with ProLong antifade mounting media containing DAPI (Molecular Probes). Images were acquired with an Olympus VS120 slide scanning microscope system using the 10x objective. Confocal images (1–2 μm optical sections) were acquired with an Olympus FV1000 laser scanning confocal microscope (Harvard Neurobiology Imaging Facility) through a 40x, 60x or 63x objective. For medial-lateral analysis of NeuN immuno and tdTomato reporter overlap in *PV* ^i-Cre^;Rosa26^lsl-tdTomato^ mice, colocalization was quantified in single plane from slide scanner images of 40 μm thick sections. The caudal-most extent of the GP was defined by the internal capsule. Each of three replicates was a different section sampling the dorsal ventral axis of the GP. For all other cell counting experiments, confocal stacks (10–30 μm in z) were obtained from sagittal sections throughout the entire medial-lateral axis of the GP. Stacks sampled the full extent of the GP (dorsal to the anterior commissure and rostral to the internal capsule) in cross section but from non-overlapping regions. For quantification of PV^+^ and PV^-^ axon territories across regions, slide scanner images were used for frontal cortex and striatum while confocal imaging was used for the STN. GFP^+^ and tdTomato^+^ pixels were binarized using conserved thresholds across replicates and the number of positive pixels counted and compared as a ratio (ImageJ). Because tdTomato exclusion from Cre^+^/GFP-expressing GP neurons was incomplete, we counted GFP^+^/tdTomato^+^ axons as GFP^+^ and subtracted those pixels to calculate the tdTomato-only fraction.

### Acute Slice Preparation

Acute brain slices were obtained from mice using standard techniques. Mice were anesthetized by isoflurane inhalation and perfused through the heart with ice-cold artificial cerebrospinal fluid (ACSF) containing (in mM) 125 NaCl, 2.5 KCl, 25 NaHCO_3_, 2 CaCl_2_, 1 MgCl_2_, 1.25 NaH_2_PO_4_ and 11 glucose (~308 mOsm·kg^-1^). Cerebral hemispheres were removed, placed in ice-cold choline-based cutting solution (consisting of (in mM): 110 choline chloride, 25 NaHCO_3_, 2.5 KCl, 7 MgCl_2_, 0.5 CaCl_2_, 1.25 NaH_2_PO_4_, 25 glucose, 11.6 ascorbic acid, and 3.1 pyruvic acid), blocked and transferred into a slicing chamber containing ice-cold choline-based cutting solution. Sagittal slices (300 μm thick) were cut with a Leica VT1000s vibratome and transferred to a holding chamber containing ACSF at 34°C for 30 minutes and then subsequently at room temperature. Both cutting solution and ACSF were constantly bubbled with 95% O_2_/5% CO_2_.

### Electrophysiology and 2-Photon Imaging

Individual slices were transferred to a recording chamber mounted on a custom built 2-photon laser scanning microscope (Olympus BX51WI) equipped for whole-cell patch-recordings and optogenetic stimulation. Slices were continuously superfused (3.5–4.5 ml·min^-1^) with room temperature ACSF or ACSF warmed to 32–34°C under feedback control (Warner Instruments). Cell-attached and current-clamp recordings were performed at 32–34°C, while voltage-clamp analysis of GP-Str connectivity was performed at room temperature. Cells were visualized through a water-immersion 60x objective using differential interference contrast (DIC) illumination. Epifluorescence illumination was used to identify cells expressing fluorescent genetic markers or GP-Str axons. In synaptic connectivity experiments, acute slices were screened for ChR2 expression in Striatum. While expression immediately adjacent to the GP was tolerated, experiments with > 200 μm striatal spread were discarded. In cases with minor leak, care was taken to record in the most dorsal and anterior striatal regions, containing ChR2^+^ axons that fit the morphology of GP-Str neurons and well away from local processes of fast-spiking interneurons at the striatal border. Patch pipettes (2–4 MΩ) pulled from borosilicate glass (G150F-3, Warner Instruments) were filled either with a Cs^+^-based internal solution containing (in mM) 120 mM CsMeSO_4_, 15 mM CsCl, 8 mM NaCl, 10 mM TEACl, 10 mM HEPES, 2 mM QX314, 4 mM MgATP, 0.3 mM NaGTP (295 MOsm; pH 7.4) for voltage-clamp and cell-attached recordings or with a K^+^-based internal solution containing of (in mM) 135 KMeSO_4_, 5 KCl, 5 HEPES, 1 EGTA, 4 Mg-ATP, 0.3 Na-GTP, 10 Na_2_-Phosphocreatine (295 MOsm; pH 7.3 adjusted with KOH) for current clamp recordings. For whole-cell recordings, Alexa Fluor 594 (10–20 μM) was added to internals to visualize cell morphology. Series resistance (<25 MΩ) was measured with a 5 mV hyperpolarizing pulse in voltage-clamp and left uncompensated. Membrane potentials were corrected for a ~7 mV liquid junction potential. After the recording was complete, cellular morphology was captured in a volume stack using 740 nm illumination from a pulsed Titanium Sapphire laser (Coherent).

### Data Acquisition and Analysis

Membrane currents and potentials were recorded using an Axoclamp 700B amplifier (Molecular Devices) filtered at 3 kHz and digitized at 10 kHz using National Instruments acquisition boards and custom software (https://github.com/bernardosabatinilab/SabalabSoftware_Nov2009.git) written in MATLAB (Mathworks). Electrophysiology and imaging data were analyzed offline using Igor Pro (Wavemetrics), ImageJ (NIH), MATLAB (Mathworks) and GraphPad Prism (GraphPad Software). In figures, voltage-clamp traces represent the average waveform of 3–6 acquisitions; current-clamp or cell-attached traces are individual acquisitions. Cell-attached recordings lasted between 3–5 minutes and were considered spontaneously active if they maintained action potential firing (>20s). Average firing rates were calculated from the whole recording period. Passive membrane properties (R_m_ and C_m_) were calculated from current deflections in voltage-clamp (V_hold_ = -77 mV). V_rest_ was calculated in current clamp from the average membrane voltage 20–60s after break in. Active membrane properties were calculated from minimum (Action Potential half-width) or maximum (FR_max_) current injections (500 ms) in current clamp. Peak amplitudes were calculated by averaging over a 1 ms window around the peak. Onset latencies for light-evoked currents were determined from the inflection points of average synaptic currents by eye.

### Statistics

Statistical analyses were performed in GraphPad Prism. Fisher’s Exact test was used to compare the fraction of GP neurons that were spontaneously active across PV genotype. The Mann-Whitney test was used to compare the firing rates of spontaneously active vs. non-spontaneously active cells across genotypes and the PSC peak amplitudes observed in PV^+^ and NPY^+^ interneurons. The means of the ratios illustrating the fraction of territory innervated by PV^+^ and PV^-^ axons by region were calculated as geometric means and compared with a Kruskall-Wallis test. P < 0.05 indicated statistical significance for all tests.

## Results

### Parvalbumin (PV) Marks Fast-Firing, Spontaneously Active Neurons in the GP

**Fig 1:**
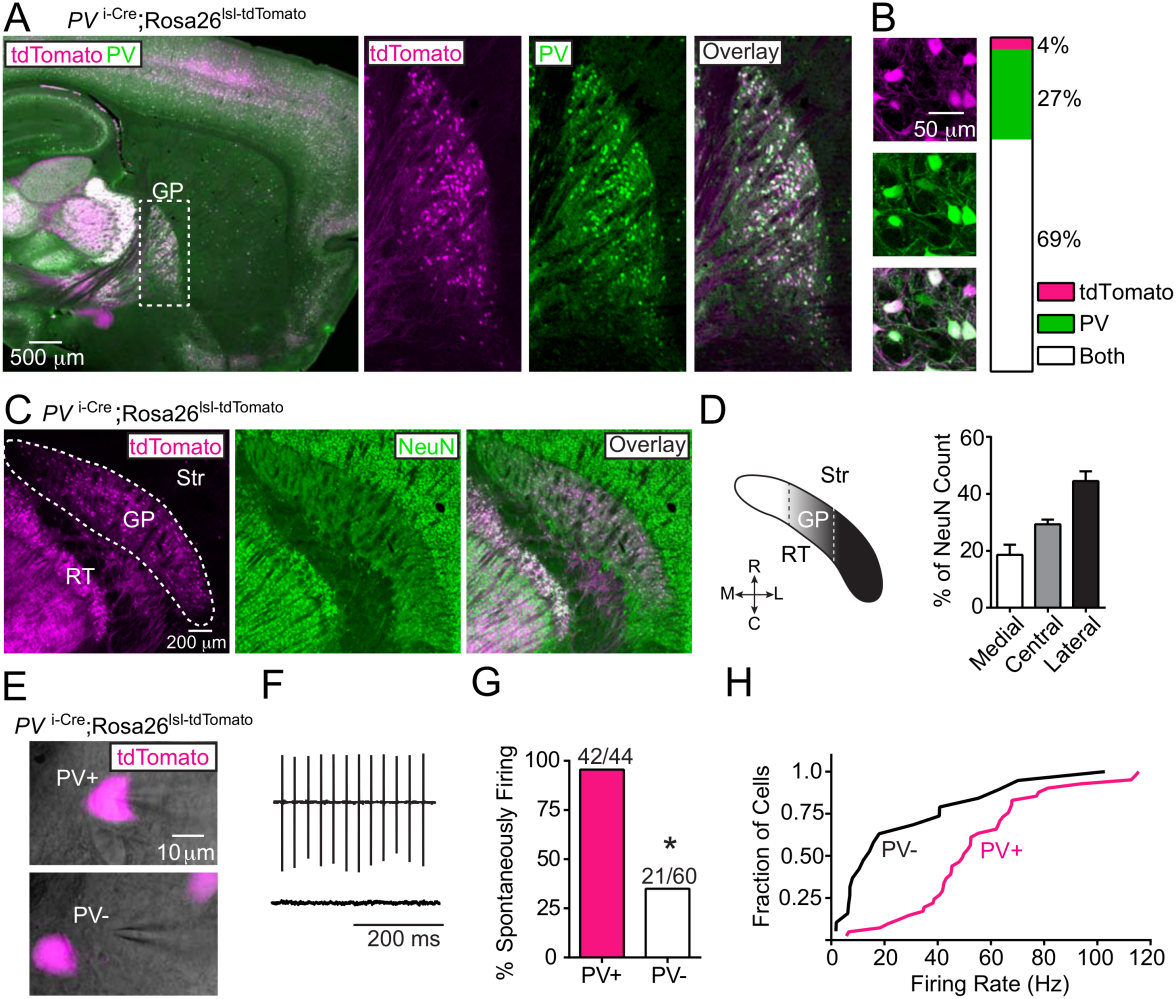
*Parvalbumin* (*PV*) expression defines a physiologically distinct cell type in the GP. (A) *Left*, sagittal brain section from a *PV* ^i-Cre^;Rosa26^tdTomato^ mouse where Cre expression is reported by tdTomato (magenta) followed by immunolabeling for PV (green). *Right*, high magnification of boxed area containing the GP. (B) Quantification of co-localization between tdTomato (magenta) and immunolabeled PV (green) in the GP using confocal microscopy. *Left*, a single confocal plane. *Right*, summary graph of co-localization results (n = 24/679 cells tdTomato^+^ only; n = 184/679 cells PV^+^ only; n = 471/679 cells double-positive, from 2 mice). (C) Horizontal section of *PV* ^i-Cre^;Rosa26^tdTomato^ mouse where Cre expression is reported by tdTomato (magenta) followed by immunolabeling for NeuN (green). (D) Quantification of the percentage of NeuN^+^ neurons with tdTomato reporter expression along the medial-lateral axis of the GP. *Left*, schematic of GP in the horizontal plane. Dashed lines delineate approximate groupings of medial, central and lateral sagittal sections. RT, reticular nucleus of the thalamus. *Right*, summary graph of mean tdTomato/NeuN percent co-localization based on sagittal sections (from 3 mice, 3 replicates/mouse). Error bars denote s.e.m. (E) Acute sagittal slice of *PV* ^i-Cre^;Rosa26^tdTomato^ mouse brain showing cell-attached recordings from tdTomato^+^ and tdTomato^-^ GP neurons. DIC image (gray) overlaid with tdTomato fluorescence (magenta). (F) Example cell-attached recordings showing action current deflections indicative of spontaneously active (*top*) and non-spontaneously active (*bottom*) GP neurons. (G) Summary graph showing the percent tdTomato^+^ and tdTomato^-^ GP neurons exhibiting cell-attached spontaneous activity (n = 29/30 *PV*^+^, n = 15/41 *PV*^-^ cells; from 3 mice). Asterisk, *P*<0.05 (Fisher’s Exact test). (H) Cumulative frequency distribution plot of firing rates from spontaneously active tdTomato^+^ and tdTomato^-^ GP neurons. Active tdTomato^+^ exhibit faster firing rates than tdTomato^-^ neurons (tdTomato^+^: mean_FR_ = 54 Hz, median_FR_ = 50; TdTomato^-^: mean_FR_ = 27 Hz; P < 0.05 Mann-Whitney Test).

To identify the cells in the GP expressing PV, we bred a transgenic mouse where Cre recombinase is transcribed under regulatory control of the endogenous *parvalbumin* locus (*PV* ^i-Cre^) and visualized Cre expression with a fluorescent reporter (Rosa26^lsl-tdTomato^). Fixed, sagittal sections of these mice revealed plentiful TdTomato^+^ neurons within the GP (Fig 1A). To test whether this transgenic approach allows genetic access without perturbing PV expression, we immunostained similar sections to test the fraction of NeuN^+^ GP neurons that are PV^+^ in wild-type and *PV* ^i-Cre^ mice. In wild-type animals (n = 3), 40% of NeuN^+^ cells (n = 6,591) also stained positive for PV^+^ (n = 2,633). In *PV* ^i-Cre^ mice, this fraction was unchanged: 40% of NeuN^+^ cells (n = 8,675) were also PV^+^ (n = 3,510). These results suggest the Cre knock-in allele does not alter PV expression in GP.

To determine whether *PV* ^i-Cre^;Rosa26^lsl-tdTomato^ mice faithfully express Cre in PV^+^ GP neurons, we performed PV immunohistochemistry using slices from reporter mice and quantified the co-localization between tdTomato^+^ and PV^+^ GP somata using confocal microscopy (Fig 1B, from 2 mice). Of n = 679 GP neurons, 69% (n = 471) were double-positive (tdTomato^+^, PV^+^), while 4% (n = 24) expressed tdTomato with no PV (tdTomato^+^, PV^-^) and 27% (n = 184) expressed no tdTomato but did immunostain for PV (tdTomato^-^, PV^+^). Thus while Cre is expressed primarily in PV^+^ neurons, these Cre^+^ neurons make up approximately 70% of the total PV^+^ GP population.

PV^+^ cell density is reported to vary along the medial-lateral axis of the both the striatum[48] and GP[25,38,39]. Using *PV* ^i-Cre^;Rosa26^lsl-tdTomato^ mice, we quantified the number of NeuN^+^ and tdTomato^+^ somata within the GP in three zones, approximating the medial, central and lateral thirds of the GP (Fig 1C and 1D). Consistent with previous reports, the mean percentage of tdTomato/NeuN cells increases along the medial to lateral axis (medial, 19%; central, 29%; lateral, 44%) with a grand average of 31% (from 3 mice). As expected from the under-labeling described above, these reporter measurements are an ~30% underestimate of the PV/NeuN percent as compared to PV immunostaining (Reporter: 31%; Immuno: 40%).

To test whether PV expression delineates functionally distinct neuron populations, we prepared acute brains slices of *PV* ^i-Cre^;Rosa26^lsl-tdTomato^ and targeted cell-attached recordings of spontaneous firing to neighboring tdTomato^+^ and tdTomato^-^ neurons (Fig 1E). To limit the effects of synaptic transmission, we included blockers of GABA_A_ (SR95331, 50 μM), AMPA (NBQX, 10 μM) and NMDA (CPP, 10 μM) receptors. Under these conditions, spontaneously active and inactive GP neurons were present and easily distinguishable (Fig 1F). While nearly 95% of tdTomato^+^ neurons were spontaneously active (n = 42/44), only 35% of tdTomato^-^ neurons fired spontaneously (n = 21/60, from 5 mice, Fig 1G). We also observed differences in spontaneous firing rates across the two populations (Fig 1H): tdTomato^+^ neurons tended to fire faster on average than neighboring tdTomato^-^ neurons (tdTomato^+^: mean_FR_ = 54 Hz, median_FR_ = 50; tdTomato^-^: mean_FR_ = 27 Hz, median_FR_ = 14; P < 0.05, Mann-Whitney test) though both populations were active over a large range (tdTomato^+^: 5.7–115.6 Hz; TdTomato^-^: 1.8–102.8). We conclude that *parvalbumin* expression defines an electrophysiologically distinct population of GP neurons, capable of spontaneous, fast-firing in acute slices. These results are consistent with the electrophysiological comparisons made by other groups [25,37,38].

### Striatal-Projecting PV^+^ GP Neurons also Innervate the Subthalamic Nucleus and Substantia Nigra

**Fig 2:**
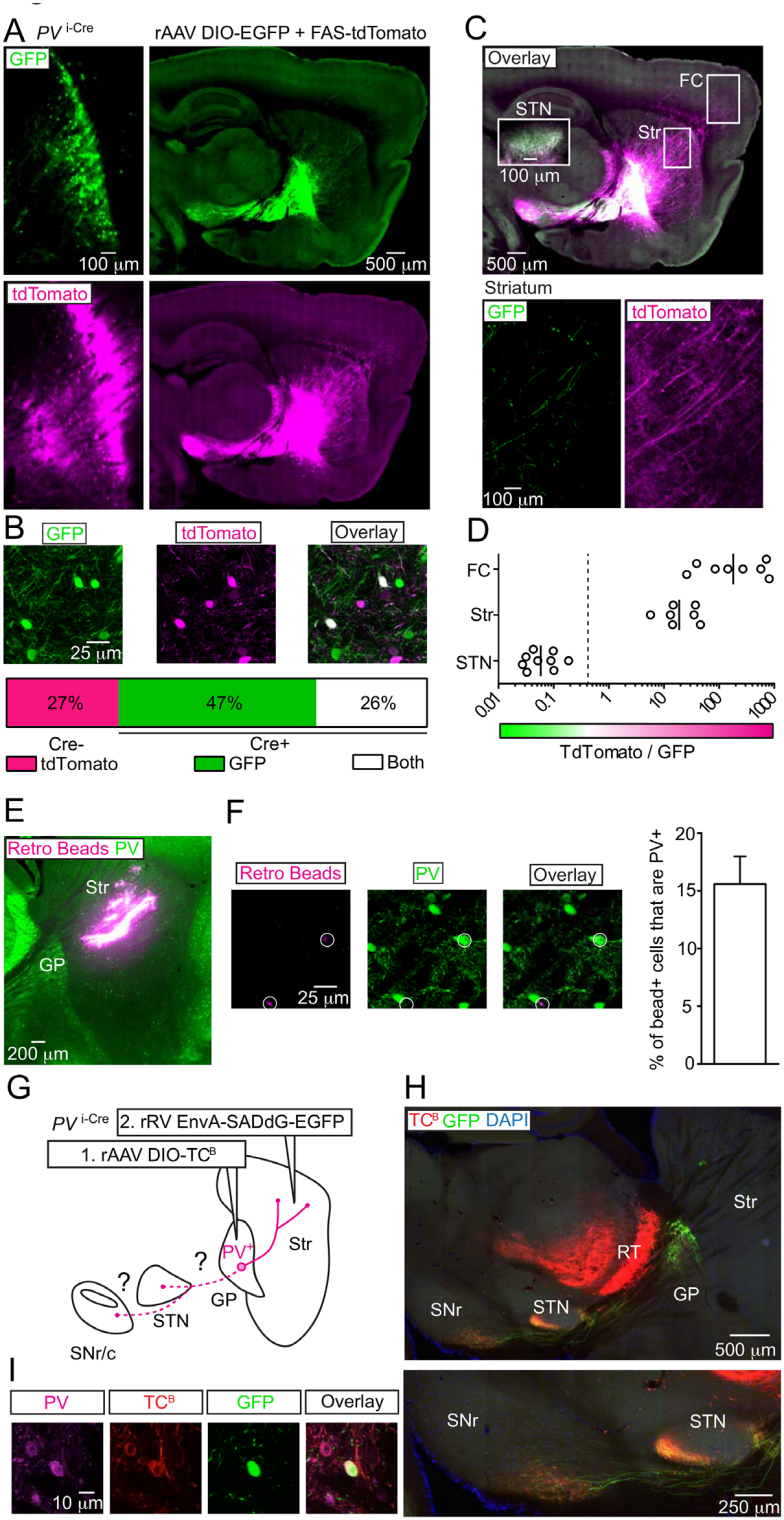
PV^+^ GP neurons innervate the striatum in addition to the STN and SNr. (A) Sagittal brain section from a *PV* ^i-Cre^ mouse injected in the GP with rAAVs DIO-EGFP (Cre-On, green) and FAS-tdTomato (Cre-Off, magenta). *Left*, injection site in GP. *Right*, low-magnification view. Look up table was changed to illustrate axonal projection patterns in low-magnification view. (B) Quantification of co-localization between GFP (green) and tdTomato (magenta) expressing GP neurons from confocal microscopy. *Top*, a single confocal plane. *Bottom*, summary graph of co-localization. All GFP expressing neurons are Cre^+^ (n = 76/285 cells tdTomato^+^ only; n = 134/285 cells GFP^+^ only; n = 75/385 cells double-positive, from n = 3 injections, from 2 mice). (C) Overlay of low-magnification sagittal sections from (A). *Top*, boxes indicate approximate regions of the frontal cortex (FC), striatum (Str) or subthalamic nucleus (STN, from an adjacent section) where GFP and/or tdTomato axonal fluorescence was quantified. *Bottom*, high magnification view of the striatum. (D) Relative expression of Cre^+^ and Cre^-^ axons in the FC, Str and STN following pixel binarization of GFP^+^ and tdTomato^+^ fluorescence. Since all GFP^+^ axons are Cre^+^, the relative abundance of Cre^-^/Cre^+^ fibers is plotted as (tdTomato-GFP)/GFP pixels. The dotted line shows the relative proportion of Cre^-^/Cre^+^ axons predicted for no selective innervation of different brain areas based on somatic co-localization quantified from the injection site (tdTomato only cells / (GFP only + tdTomato and GFP double-positive cells), from data in (B): 76/(134+76) = 0.364). Solid lines represent geometric means (n = 3 injections, from 2 mice). Innervation ratios were different across regions (P < 0.05, Kruskall-Wallis test). (E) Retrograde labeling of PV^-^ and PV^+^ GP somata. Sagittal section of wild-type mouse following injection of retro beads (magenta) in dorsal striatum and immunolabeling of PV (green). (F) Quantification of co-localization between retro bead^+^ (magenta) and PV^+^ (green) cells in the GP using confocal microscopy. *Left*, a single confocal plane. Bead^+^ cells are highlighted with circles. *Right*, summary graph of mean co-localization (from 4 mice). Error bars denote s.e.m. (G-I) Full anatomical visualization of PV^+^ GP-Str neurons following rabies-based retrograde labeling in *PV* ^i-Cre^ mice. (G) Experimental strategy to test whether PV^+^ GP-Str neurons also innervate the STN and SNr. First, rAAV DIO-TC^B^ is injected into the GP of *PV* ^i-Cre^, allowing TC^B+^ axons to be selectively transduced by EnvA pseudo-typed recombinant rabies virus (rRV). Second, replication-incompetent rRV EnvA-SADdG-EGFP is injected into the striatum 2 weeks after the rAAV injection. GFP^+^ axons in the STN and SNr indicate innervation by PV^+^ GP-Str neurons. (H) *Top*, low magnification sagittal section from an example double-recombinant viral experiment described above. TC^B+^ expression is restricted to Cre^+^ neurons of the GP and reticular nucleus of the thalamus (RT). The vast majority of GFP^+^ somata were observed in the GP. Additionally, a sparse number of GFP^+^ somata with glial morphology or aspiny dendrites were also present in the striatum. GFP^+^ axons were clearly visible in the striatum, as well as the STN and SNr. *Below*, higher magnification view of the STN and SNr from the section above. Similar labeling was observed in n = 6/6 injections from 3 mice and n = 0/4 control injections from 2 mice in which DIO-TC^B^ was replaced by DIO-mCherry. (I) A single confocal plane of the GP from the experiment shown, demonstrating that TC^B+^/GFP^+^ GP neurons are also immuno-positive for PV.

To compare the axonal innervation patterns of PV^+^ and PV^-^ GP neurons, we injected two recombinant adeno-associated viruses (rAAVs) into the GP of *PV* ^i-Cre^ mice designed to selectively express different fluorophores in intermingled Cre^+^ and Cre^-^ populations[46]. Following injection DIO-GFP (Cre-activated) and FAS-tdTomato (Cre-inactivated) rAAVs into the GP, we observed GFP and tdTomato expression in GP somata and axons throughout the BG and cortex (Fig 2A). No retrograde labeled somata were observed in these areas, consistent with the limited retrograde labeling of serotype 8 rAAVs[49]. While GFP expression is only activated in Cre^+^, tdTomato is expressed in all neurons before Cre-mediated excision inactivates FAS rAAV transcription. Thus efficient exclusion of tdTomato from Cre^+^ neurons is dependent on the relative concentrations of FAS rAAV genomes and Cre in the nucleus.

To evaluate the use of this system in the GP of *PV* ^i-Cre^ mice, we quantified co-expression of GFP and tdTomato within individual GP neurons using confocal microscopy (Fig 2B). Of n = 285 imaged cells, 27% (n = 76) expressed tdTomato with no GFP (tdTomato^+^ GFP^-^), 47% (n = 134) expressed GFP alone (tdTomato^-^ GFP^+^) and 26% (n = 75) were positive for both tdTomato and GFP (tdTomato^+^ GFP^+^, from 2 mice). The presence 26% double-positive neurons indicate that Cre molecules were not abundant enough to efficiently inactivate tdTomato expression, as robust Cre inactivation has been observed in other Cre transgenic mice[28]. Nevertheless, the ratio of Cre^-^/ Cre^+^ somata in these experiments (76/(134+75) = 0.36) can be compared to the ratio of Cre^-^/ Cre^+^ axonal territories to estimate innervation biases emerging from PV^-^ or PV^+^ GP neurons.

To estimate preferential innervation of frontal cortex, striatum and subthalamic nucleus by PV^+^ or PV^-^ GP axons, we quantified Cre^-^/Cre^+^ axonal territories within each of region (Fig 2C). In frontal cortex and striatum, axon densities were quantified from slide-scanner images of 40 μm thick tissue sections; In the STN, confocal stacks were used because of the higher densities of axons. GFP^+^ and GFP^-^/tdTomato^+^ pixels were then binarized using conserved thresholds and counted. We report the ratios of those positive-pixel counts. Since FAS-tdTomato expression was also present in the striatum immediately adjacent to the GP, we excluded the SN_r/c_ from this analysis because of contamination from tdTomato^+^ dSPN axons. In all three regions examined, the Cre^-^/Cre^+^ axonal fraction differed markedly from 0.36, the null expectation of no selective innervation (Fig 2D). While the PV^+^ GP fraction preferentially arborized in the STN (geometric mean ±[95%CI], 0.06 [+0.1, -0.04]), the PV^-^ fraction tended to arborize extensively in the striatum (19.2 [+38.4, -9.6]) and even more extensively in the cortex (180.4 [+548.9, -59.3])(P < 0.05, Kruskall-Wallis test), similar to previous observations[25]. The adjacent reticular nucleus of thalamus (RT) was innervated by axons expressing tdTomato but not GFP, suggesting the PV^+^ GP neurons do not make a significant RT projection as observed previously[25].

This anterograde labeling suggests that PV^+^ neurons make up the minor population of GP neurons that project to striatum (GP-Str). To quantify the fraction of GP-Str neurons that express PV, we retrogradely labeled ipsilateral GP-Str neurons by injecting retro beads in dorsal striatum and immunostained the bead-labeled tissue for PV (Fig 2E). We measured co-localization between bead^+^ and PV^+^ GP somata using confocal microscopy (Fig 2F). On average, 17% of GP-Str cells were PV^+^ (n = 50/296, from 4 mice), confirming that GP neurons immunopositive for PV make up a pronounced yet minor population of GP-Str neurons. This proportion of PV^+^ pallidostriatal GP neurons is similar to that observed through retrograde viral tracing in rats [37].

To determine whether PV^+^ GP-Str neurons innervate other nuclei of the BG, we used a double-recombinant viral strategy to visualize axonal morphology through retrograde labeling (Fig 2G). First, we injected rAAV DIO-TC^B^ into the GP of *PV* ^i-Cre^, driving an enhanced version of the TVA receptor fused to mCherry (TVA-mCherry^Bright^ or TC^B^) [50] exclusively in Cre^+^ neurons of the GP and the adjacent reticular nucleus of thalamus (RT). Second, we injected the striatum of these mice with a replication incompetent strain of recombinant rabies virus, pseudotyped with EnvA and expressing EGFP (EnvA-SADdG-EGFP). Since EnvA^+^ rRV require TVA for endocytosis, only TC^B+^ axons should be transduced. As expected, we observed GFP^+^ axons in the striatum emanating from GP neurons that were expressing TC^B^ (Fig 2H and 2I). Confocal imaging demonstrated that of n = 32 GFP^+^ GP neurons, n = 31 (or 97%) were detectably TC^B+^. These TC^B^ cells were also immune-positive for PV (82% of TC^B^ cells were PV^+^, n = 286/347). GFP^+^ axons also made prominent arborizations in the STN and SNr, demonstrating that at least a subset of PV^+^ GP-Str innervate other BG nuclei (observed in n = 6/6 injections in 3 mice). We do not believe RT leak contributed to the rabies tracing as 1) RT neurons are not known to innervate the striatum and 2) no somatic GFP expression was observed in the RT. We confirmed that retrograde labeling was genetically targeted, as *PV* ^i-Cre^ mice injected with rAAV DIO-mCherry in GP and thus lacking TVA showed no GFP expression following transduction rRV EnvA-SADdG-EGFP (n = 0/4 injections in 2 mice). In the TC^B^ mice, we observed a small number of GFP^+^ cells in the striatum that were absent in mCherry controls. These cells had either 1) glial morphology or 2) aspiny dendrites and thus were unlikely to project an axon outside of the striatum.

### PV^+^ GP Neurons Innervate Distinct Types of Striatal Interneurons

The function of PV^+^ GP neurons in controlling activity within the BG depends on the identities of the post-synaptic cell and activated synaptic receptors. Using a combination of transgenic markers and 2-photon imaging of neuronal morphology in acute brain slices, we devised a strategy to identify each of the five major striatal cell types: the direct or indirect pathway spiny projection neurons (dSPNs and iSPNs) and the three major interneuron classes—low-threshold spiking (LTS), fast-spiking (FS) and choline acetyltransferase expressing (ChAT).

**Fig 3:**
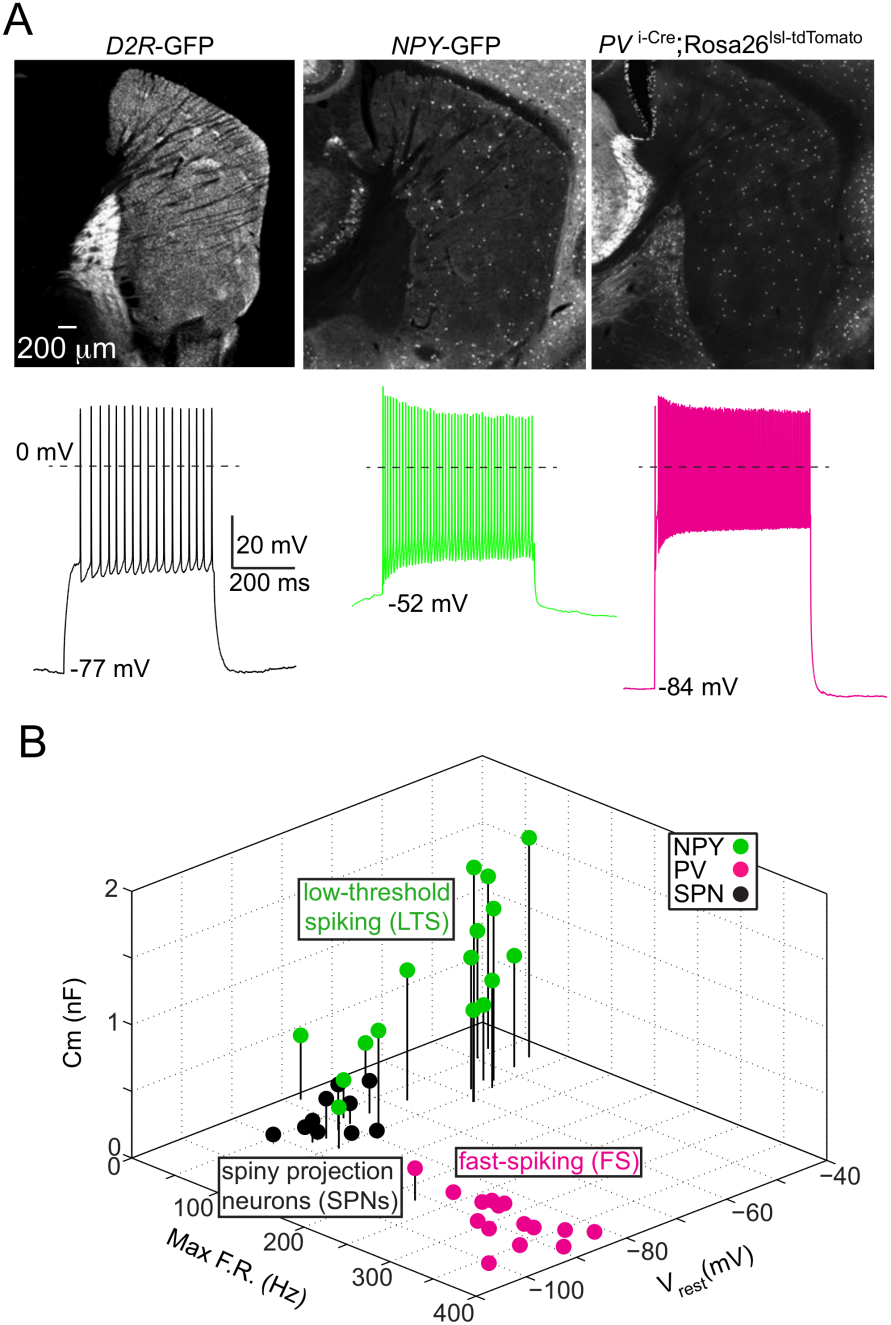
Molecular markers distinguish low-threshold spiking (LTS) and fast-spiking (FS) striatal interneurons from spiny projection neurons (SPNs). (A) *Top*, sagittal brain sections from transgenic mice used to distinguish striatal cell types through selective expression of fluorescent proteins. The dopamine 2 receptor BAC (*D2R*-EGFP) drives EGFP selectively iSPNs, while the Neuropeptide Y BAC (*NPY*-EGFP) drives EGFP selectively in putative NPY^+^ LTS interneurons and a smaller number of NPY^+^ neurogliaform interneurons[51]. The *PV* ^i-Cre^;Rosa26^lsl-tdTomato^ mouse expresses tdTomato in *PV*^+^ putative FS interneurons. *Bottom*, example membrane potential recordings for each fluorescently labeled cell type in response to current injections (500 ms, SPN: 200 pA; LTS: 400 pA; FS: 1500 pA). (B) Three-dimensional scatter plot of membrane capacitance (C_m_), maximum firing rate (Max FR) and resting membrane potential (V_rest_) for striatal cell types color-coded by expressed molecular marker.

SPNs have distinctively spiny dendrites and can be pathway-classified based on the expression of GFP under the dopamine 2 receptor regulatory elements (*D2R*-GFP^+^, iSPNs; *D2R*-GFP^-^, putative dSPNs). LTS interneurons express Neuropeptide Y, whereas FS interneurons express parvalbumin[1]. Thus each cell type should be differentially marked in mice where fluorophores are under regulatory control of the NPY (*NPY*-GFP^+^, LTS) or parvalbumin locus (*PV* ^i-Cre^;Rosa26^lsl-tdTomato^, FS). Choline acetyltransferase (*ChAT*) interneurons have large somata and do not require a molecular marker for identification. Indeed, each of the three transgenes marks cells with the expected striatal anatomy (Fig 3A).

To determine if the transgenic alleles mark neurons with the expected electrophysiological profiles, we targeted whole-cell recordings to fluorophore-expressing striatal neurons in acute slices of *NPY*-GFP and *PV* ^i-Cre^;Rosa26^lsl-tdTomato^ mice. We measured passive and active membrane properties in voltage- and current-clamp using a K^+^-based internal solution and compared these values for each marked cell type, as well as non-fluorophore expressing neighboring SPNs (Fig 3A). *NPY*-GFP^+^ and tdTomato^+^ neurons were clearly distinguished by electrophysiological properties (Table 1). A scatter plot based on 3 parameters—maximum firing rate, membrane capacitance and resting membrane potential—revealed that the 3 genetically marked populations fell into 3 electrophysiological clusters, suggesting *NPY*-GFP and *PV* ^i-Cre^ expression faithfully marks LTS and FS interneurons respectively (Fig 3B). The n = 2 *NPY*-GFP^+^ neurons grouped within the SPN cluster may correspond to the minor interneuron population of *NPY*^+^ neurogliaform cells, which have lower membrane resistance and resting potential [51].

**Table 1:** Electrophysiological properties of striatal cell types.

Final			
	*NPY*	*PV*	SPN
*N*	16	16	10
AP half-width (ms)	1.3 ± 0.1	0.47 ± 0.03	1.2 ± 0.09
FRmax (Hz)	96 ± 8	322 ± 12	99 ± 5
Vrest (mV)	-65 ± 3.2	-91 ± 1.3	-87 ± 1.6
Holding current at -77 mV (pA)	-24 ± 5	157 ± 28	58 ± 16
Input resistance (MOhm)	970 ± 113	92 ± 19	248 ± 36
Capacitance (pF)	64 ± 7	88 ± 4	130 ± 13

**Fig 4:**
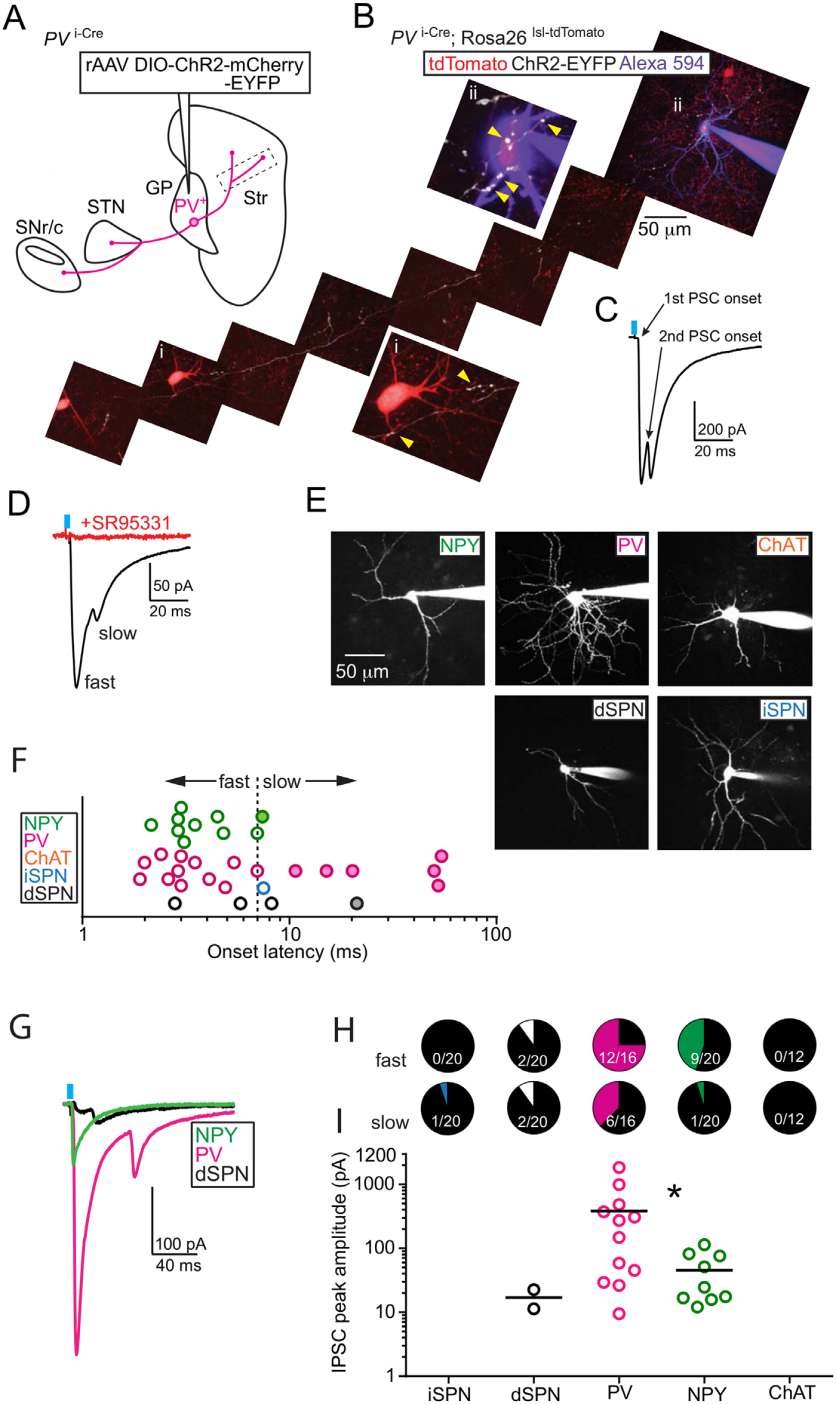
Striatal-projecting axons from *PV*^+^ GP neurons primarily innervate fast-spiking (FS) and low-threshold spiking (LTS) interneurons. (A) Schematic of strategy to target ChR2 to *PV*^+^ GP neurons. DIO rAAVs expressing Cre-On ChR2 fused to either mCherry or EYFP were injected into the GP of *PV* ^i-Cre^ mice, leading to ChR2^+^ axons in the striatum, STN and SNr. Dashed box shows approximate location of *PV*^+^ GP-striatal axon in (B). (B) Maximum projection 2-photon stacks of a ChR2-EYFP^+^ axon (white) imaged from an acute slice of *PV* ^i-Cre^;Rosa26^lsl-tdTomato^ striatum following whole-cell recording and dialysis of tdTomato^+^ (red) interneuron with Alexa Fluor 594 (purple). Insets show high-magnification views of putative ChR2^+^ pre-synaptic terminals (yellow arrowheads) onto proximal dendrites and somata of un-recorded (i) and recorded (ii) tdTomato^+^ interneuron. (C) Light-evoked post-synaptic current (PSC) recorded in voltage-clamp (V_hold_ = -77 mV) from interneuron shown in (B). Arrows indicate onsets for the 1^st^ and 2^nd^ PSC peaks. (D) Example of light-evoked PSCs from a *PV*^+^ fast-spiking interneuron. This current has both a fast and slow peak and is blocked by bath application GABA_A_ receptor antagonist SR95331 (50 μM). (E) Maximum projection 2-photon stacks of five striatal cell types following whole-cell recording and dialysis of Alexa Fluor 594. Cell types are identified through the expression of molecular markers and/or the morphology of somata and dendrites. (F) Onset latencies for all recorded light-evoked PSC peaks color-coded by cell type. Open circles represent PSCs with a single peak. Filled circles represent the second PSC peak in cells with two peaks. PSCs that occurred ≤7 ms were classified as “fast”, >7 ms as “slow”. 7 ms represents the fastest “slow” (putative polysynaptic) response observed (green filled circle). (G) Examples of light-evoked PSCs by cell type. (H) Pie-charts illustrating the percentage of recorded neurons by cell type exhibiting either fast or slow PSC. Fractions illustrate the number of cells in which a PSC was detected over the total number of cells recorded. (I) Peak amplitudes for all fast PSCs by cell type. Black bars indicate mean values. Mean (± s.e.m) PSC peaks are larger in PV^+^ interneurons (-384 ± 156 pA) than NPY^+^ interneurons (-46 ± 12 pA). Asterisk, P < 0.05 (Mann-Whitney Test).

Having validated our system for identifying striatal cell types, we sought to determine if PV^+^ GP-Str axons inhibit post-synaptic neurons through GABA_A_ receptors. Since GP-Str cells have been shown to anatomically target PV^+^ cells in rat striatum[52], we focused initially on striatal FS interneurons. To visualize PV^+^ interneurons and optogenetically activate PV^+^ GP-Str axons, we introduced the light-activated ion channel channelrhodopsin2 (ChR2) fused to EYFP into the GP of these *PV* ^i-Cre^; Rosa26^lsl-tdTomato^ mice through intracranial injection of a Cre-activated rAAV (DIO-ChR2-EYFP, Fig 4A). After >2 weeks, we cut acute brain slices and observed ChR2^+^ axons coursing from the GP into the striatum, making bouton-like varicosities that made contact with the somata and proximal dendrites of tdTomato^+^ neurons (Fig 4B). To test whether these varicosities correspond to functional inhibitory synapses, we used a Cs-based internal solution (calculated E_Cl_ = -36 mV) to make whole-cell voltage clamp recordings (V_hold_ = -77 mV) from tdTomato^+^ somata surrounded by ChR2^+^ axons. Recordings were made in the presence of antagonists for AMPA- (NBQX, 10 μM) and NMDA- (CPP, 10 μM) type glutamate receptors to isolate GABAergic transmission. Brief pulses of blue (473 nm) light (2 ms, 1.3 mW x mm^-2^) evoked inward post-synaptic currents (PSCs), consistent with an outward flux of Cl^-^ ions through GABA_A_ receptors (Fig 4C). PSCs often had two peaks (Fig 4C) and were abolished by bath application of SR95331 (50 μM), confirming their dependence on GABA_A_ receptors (Fig 4D). We conclude that PV^+^ GP-Str axons activate ionotropic GABA_A_ receptors through the release of GABA.

To assess whether PV^+^ GP-Str neurons make inhibitory synapses with the other types of striatal neurons, we bred additional *PV* ^i-Cre^ mice carrying either the *D2R*-GFP or *NPY*-GFP allele. Following injection of rAAV DIO-ChR2-mCherry in the GP, connectivity was assayed as above, targeting recordings to somata within <100 μm of ChR2^+^ axons. We excluded experiments containing slices with ChR2 expression > 200 μm into the striatum from the GP border. In cases with minor leak, we recorded in the anterior and dorsal most regions of striatum, well away from local interneuron processes. Following whole-cell recording and dialysis with Alexa Fluor 594, cell type was confirmed by imaging neuronal morphology (Fig 4E).

Light activation evoked PSCs in all cell types (iSPNs and putative dSPNs, FS and LTS interneurons) except ChAT interneurons. The PSC onset latency ranged from 1.9–8.2 ms and was sometimes interrupted by a 2^nd^ PSC with onset latency from 7.4–54.3 ms, suggesting light activation stimulated both mono and poly synaptic inputs (Fig 4F). We did not attempt to disambiguate mono and poly synaptic inputs experimentally. We classified PSCs with onsets <7ms as “fast” and those >7ms as “slow,” based on the assumption that, in cells with two PSC peaks, the 2^nd^ PSC represents a polysynaptic input. We used 7ms as a cut off because it represents the onset of the shortest-latency 2^nd^ PSC we observed (Fig 4C and 4D). Polysynaptic inputs could result from post-inhibition rebound firing of neurons within the striatum or the GP, although the orientation of the slice largely severs GP axons in the striatum from their somata.

Our connectivity analysis revealed clear differences in light-evoked PSCs by cell type (Fig 4G–4I). PV^+^ FS and presumed NPY^+^ LTS interneurons had the highest rates of connectivity, with fast PSCs detectable in >40% of recorded cells. PSCs in FS and LTS were not equivalent: FS cells tended to have larger PSCs than LTS cells (mean: -437.2 vs. -46.5 pA, P<0.05 Mann-Whitney) and more frequent “slow” PSCs (n = 6/16 vs 1/20, P = 0.095, Fisher’s Exact Test). “Fast” PSCs were detected in a small number of putative dSPNs (n = 2/20) but never in iSPNs. These “fast” PSCs in putative dSPNs had smaller peaks (mean: -12.3 pA) as compared to those recorded from FS and LTS interneurons.

## Discussion

We sought to understand how PV^+^ GP neurons fit into a cell-type based model of BG circuits, using transgenic mice and recombinant viruses to selectively label and light-activate this neuronal population. We focused on the contribution that PV^+^ GP neurons make to the pallidostriatal (GP-Str) projection. Our results indicate that PV^+^ GP neurons contribute a minor fraction of GP axons in the striatum (~17%), consistent with previous reports in rodents [25,37,38,40,53]. PV^+^ neurons are intrinsically spontaneously active, firing at 54 Hz on average. Neighboring PV^-^ neighbors, when spontaneously active, tend to fire at slower average rates (27 Hz). These differences in spontaneous firing rate may contribute to the LFP in the GP, which exhibits peaks at both 14 and 60 Hz[54]. PV^+^ GP-Str cells provide potent and robust (likely monosynaptic) GABAergic inputs onto PV^+^ FS and NPY^+^ LTS striatal interneurons and weak and infrequent inputs onto dSPNs. Some PV^+^ GP-Str neurons also innervate the STN. This connectivity suggests PV^+^ GP-Str cells are positioned to directly coordinate firing of STN neurons and PV^+^ FS and NPY^+^ LTS striatal interneurons through monosynaptic inputs. PV^+^ GP-Str stimulation also evoked polysynaptic inhibitory PSCs in the striatum, most frequently in *PV*^+^ FS interneurons. Since the vast majority of GP-Str axons were likely severed in our slice preparation, and glutamate receptors were pharmacologically blocked, these PSCs might be driven by non-excitatory rebound firing within the striatum. We note that definitively determining mono vs polysynaptic innervation—and the effects of these inputs on striatal microcircuitry—require further investigations.

Despite the paucity of PV^+^ neurons contributing to the GP-Str projection, these cells appear to have unique electrophysiological properties and synaptic organization within the BG as compared to canonical Arkypallidal cells. Furthermore, within-striatum connectivity also appears to be different, as Arkypallidal cells are poised to innervate SPNs and ChAT interneurons[40]. These synaptic differences complement differences in electrophysiological characteristics and suggest that GP-Str PV^+^ cells may be a distinct cell type within the larger PV^+^ class.

What function do PV^+^ GP-Str neurons play in coordinating activity within the recurrent, looped circuitry that interconnect the striatum and GP? SPNs are presumed to be capable of inhibiting spontaneously active GP neurons [19]. These pauses in GP firing are thought to be an important component of BG neural codes [55]. Currently, it is unclear whether iSPNs, dSPNs and the STN differentially innervate Arkypallidal, Prototypic and the non-canonical pallidostriatal PV^+^ GP neurons described here. Defining the circuit effects of these synaptic interactions will be important for understanding the role of the GP during behavior. Other GP cell types, such as the GP-FC cells, are inhibited by both iSPN and dSPNs, but with different strengths and short term synaptic properties[28]. These results suggest that the dynamics of striatal-induced pausing of the GP may depend on both the pre and post-synaptic cell types. If meaningful, these cell type interactions could allow the GP to function as a synaptic “switchboard,” where SPN activity has disparate effects on GP cell types with different projection patterns. Future experiments combining pathway specific optogenetic activation with recording from identified GP neurons should address these possibilities.

Feedback from the GP likely regulates striatal processing in a cell type specific manner. PV^+^ GP-Str cells directly control striatal activity primarily via inhibitory inputs on FS and LTS interneurons. dSPNs and iSPNs are strongly inhibited by neighboring FS cells, whereas inhibition from LTS cells may be more spatially dispersed [56]. To a first approximation, the potent inhibition of FS and LTS cells by PV^+^ GP-Str axons should disinhibit SPNs of both pathways. However, the direction of this effect could depend on the synaptic properties. For example, maintained gamma rate (20–80 Hz) firing could depress PV^+^ GP-Str synapses onto interneurons, providing little sustained inhibitory conductance. In this scenario, inhibition of striatal interneurons would be most effective following a pause in PV^+^ GP-Str activity. Additionally, the efficacy of PV^+^ GP-Str inhibition may depend on the concerted activity of multiple synaptic inputs. Counterintuitively, highly synchronized inhibition may permit firing, as exhibited by other spontaneously active GABAergic circuits operating at fast-firing rates [57].

Canonical PV^-^ Arkypallidal cells, which make up the bulk of the pallidostriatal projection, likely control striatal feedback through different cell types and synaptic properties. Anatomical evidence suggests that PV^-^ GP-Str cells also innervate SPNs and ChAT interneurons in addition to those FS and LTS cells targeted by the PV^+^ pallidostriatal system[25,40]. Arkypallidal cells seem poised to regulate striatal output in both mono and polysynaptic inhibition. The slower, more irregular firing of Arkypallidal cells also suggests that their inhibitory synapses may function in a different regime than the more regular, fast spiking PV^+^ counterparts. Future optogenetic studies of these various synaptic inputs should help highlight the functional differences between these two pallidostriatal cell types.

Much of the coordinated timing necessary for BG function is thought to arise through the synaptic interactions of the GP and STN, yet the diversity within the contributing cell types remain largely undescribed. The STN contains spontaneously active glutamatergic neurons, which through their reciprocal synapses with GP, create a network with intrinsic firing rate oscillations. The integrity of these oscillations is believed to be necessary for normal BG function[32]. In current BG models, the GP-STN oscillator affects the striatum indirectly, via local interactions in the GP or through the BG-thalamus-cortex or BG-cortex[28,29] loops. In this study, we show that at least a subset of PV^+^ GP-Str cells also innervate the STN. This neuronal population provides a circuit for direct coordination of striatal interneurons and the STN.

Our results advance our understanding of how PV^+^ GP neurons contribute to BG circuits. We confirm that PV^+^ cells are 40% of the mouse GP and reveal how the pallidostriatal subset of this population is positioned to directly influence neural processing in the striatum and STN, the major BG input nuclei. Canonical Prototypic and Arkypallidal neurons exhibit many unique features in the healthy and dopamine depleted brain and future work will reveal whether PV^+^ GP-Str cells are more Prototypic or Arkypallidal in nature. Direct molecular characterization of PV^+^ GP-Str cells will help address this question and determine whether these cells are accessible through intersectional genetics. Candidate markers include Lhx6 and/or Npas1, transcription factors which have shown variable expression across Prototypical/Arkypallidal classes in studies using different rodents[37,38]. Large-scale single-cell transcriptional assays could also help identify the molecular make up of PV^+^ GP-Str cells in addition to providing an unbiased assessment of GP cell diversity[58]. Lastly, future synaptic and anatomical analyses are needed to define the sources and properties of synapse that control Prototypic cells, Arkypallidal cells, and the PV^+^ GP-Str neurons described here. These data are necessary to fully orient these cell types within the circuitry of the BG. Ensuing connectivity models of the GP will provide an invaluable framework for testing how particular GP cell types control activity across the BG, thalamus and cortex during behavior.
